# The Use of Bioactive Polymers for Intervention and Tissue Engineering: The New Frontier for Cardiovascular Therapy

**DOI:** 10.3390/polym13030446

**Published:** 2021-01-30

**Authors:** Francesco Nappi, Antonio Nenna, Domenico Larobina, Giorgia Martuscelli, Sanjeet Singh Avtaar Singh, Massimo Chello, Luigi Ambrosio

**Affiliations:** 1 Department of Cardiac Surgery, Centre Cardiologique du Nord de Saint-Denis, 93200 Paris, France; 2 Department of Cardiovascular Surgery, Università Campus Bio-Medico di Roma, 00128 Rome, Italy; a.nenna@unicampus.it (A.N.); m.chello@unicampus.it (M.C.); 3 Institute for Polymers, Composites and Biomaterials, National Research Council of Italy, 06128 Rome, Italy; domenico.larobina@cnr.it (D.L.); luigi.ambrosio@cnr.it (L.A.); 4 Multidisciplinary Department of Medical-Surgical and Dental Specialties, University of Campania Luigi Vanvitelli, 81100 Naples, Italy; martuscelligiorgia@gmail.com; 5 Department of Cardiothoracic Surgery, Golden Jubilee National Hospital, Glasgow G81 4DY, UK; sanjeetsa_singh@gmail.com

**Keywords:** drug-eluting stent, polymers, bioresorbable scaffolds, coronary, cardiovascular

## Abstract

Coronary heart disease remains one of the leading causes of death in most countries. Healthcare improvements have seen a shift in the presentation of disease with a reducing number of ST-segment elevation myocardial infarctions (STEMIs), largely due to earlier reperfusion strategies such as percutaneous coronary intervention (PCI). Stents have revolutionized the care of these patients, but the long-term effects of these devices have been brought to the fore. The conceptual and technologic evolution of these devices from bare-metal stents led to the creation and wide application of drug-eluting stents; further research introduced the idea of polymer-based resorbable stents. We look at the evolution of stents and the multiple advantages and disadvantages offered by each of the different polymers used to make stents in order to identify what the stent of the future may consist of whilst highlighting properties that are beneficial to the patient alongside the role of the surgeon, the cardiologist, engineers, chemists, and biophysicists in creating the ideal stent.

## 1. The Clinical Problem

Coronary heart disease (CHD) is one of the leading causes of death in many countries, as it is estimated that each minute, a myocardial infarction (MI)-related death occurs [1]. Other than first or recurrent symptomatic CHD registered as hospitalized MI or cardiac-related deaths, about 20% of events still remain silent [2,3]. Each year, scientifical societies estimate 515,000 new attacks and 205,000 recurrent attacks, with an average first presentation age of 64.9 years for men and 72.3 years for women [2,4,5,6]. Data from the Framingham Heart Study (FH) revealed that CHD constitutes more than half of all cardiovascular events in the aged population [2,3]. Fortunately, early reperfusion strategies (percutaneous coronary intervention, PCI) and the decline in ST-elevation myocardial infarction (STEMI) presentation (from 133 to 50 cases per 100,000 person/years) have reduced CHD-associated mortality rates. In this setting, nearly two million stents are used annually (Interventional Cardiology Devices Market Report Suite for US, 2018–2024, available at https://idataresearch.com/product/interventional-cardiology-market/) for CHD [5]. However, stent thrombosis remains a significant complication and is generally associated with adverse clinical events [4,7]. The targeted delivery of drugs in coronary atherosclerotic disease was an inviting avenue, with early bare-metal stents (BMSs) gradually replaced by drug-eluting stents (DESs) [4]. The advantages of localized drug delivery, affecting, through a paracrine mechanism, the sites of disease, were established as the ideal strategy for handling coronary artery disease (CAD) in patients with lipid dysmetabolic disease and restenosis [2,4,5]. However, some patients have more complex vascular lesions, and the use of targeted delivery of drugs may be ineffective with potentially harmful side effects [8]. Neo-atherosclerosis is not an infrequent event in patients who received a DES [7]. Unstable features of neo-atherosclerosis, even though identified in both BMSs and DESs, appear to be related to shorter durability only for DESs [9,10,11,12]. The development of neo-atherosclerosis may represent another rare factor contributing to the onset of late thrombotic events [7].

This review intends to summarize the historic development and technologic challenges of stents, from first-generation bare-metal stents to newer devices, with a comprehensive and a translational outlook. Current results of major clinical trials will be discussed along with the advantages and disadvantages offered by each of these different polymers used to make newer stents. Current-generation stents represent a milestone for future development and clinical use.

## 2. From Bare-Metal Stents to Absorbable Stents: The Evolutionary Phase of Percutaneous Coronary Intervention

The higher rate of restenosis with clinically relevant adverse events (acute MI or unstable angina) has been associated with BMSs by Chen et al [13]. This raised concerns regarding the neo-atheromatous development as a pathophysiological mechanism that could cause plaque rupture within the neointima. The use of a stent incorporating the ability to deliver a drug was seen as the panacea to prevent the risk of restenosis. Holmes et al. [14] remarked that the novel percutaneous device to treat CHD was “only the most immediate and obvious example of a major paradigm shift” in the field of cardiological science. DESs were presented as the solution to the problem of restenosis that occurred in BMSs which had been used to re-establish blood flow due to the occurrence of target coronary lesions since the 1980s.

The successful incorporation of a drug in a stent depends on the assimilation of techniques, with an interdisciplinary collaboration between cardiologists, engineers, chemists, and biophysicists. Many factors had to be taken into consideration to optimize the functioning of these devices. One of the most important critical issues is the release kinetics, which is fundamental to obtain maximum efficacy of the drug while minimizing adverse effects. The work must therefore be oriented towards the following lines. First of all, the properties of the drug, stent, and coating material are crucial [15], with a specific interest in its thickness. Secondly, the drug delivery mechanism and the initial concentration placed on the stent [15,16] alongside the geometry of the stent [17] must be considered. Ultimately, fluid dynamics must guide research towards stent-induced changes in blood flow patterns [17] and the potential development of a thrombus in and around the stent [18] as well as the possible diffusion and consequent absorption of the drug in the surrounding tissue [19].

Coronary stent implantation significantly affects blood fluid dynamics. Assuming that no “defects” occur, either during the stent implantation (malapposition) or as a result of stent degradation (scaffold repositioning) later on, the main source of fluid dynamic changes is the stent struts [7]. In designing a stent platform, it is, therefore, crucial to correct the dimensions around this parameter. Numerical hemodynamic simulations play a pivotal role in determining the main issues of stent/scaffold failure, i.e., restenosis, device thrombosis, and neo-atherosclerosis. Those problems are related both to the presence of stagnation points and to changes in the spatial and temporal distribution of shear stress at the wall (WSS). In fact, the literature notes that stagnation points can be related to stent thrombosis, and the distribution of WSS can induce a high inflammatory reaction through mechanotransduction pathways, ultimately leading to restenosis. In simulating the fluid dynamics around a stent, all components of the device must be properly taken into account, including the presence of a polymer coating or the release of a drug. Indeed, if the stent is coated with a polymer, the interfacial interaction between the blood and the polymer can play an important role in re-determining the hemodynamics. Finally, we recall that the margination of blood components (red blood cells, white blood cells, and platelets) plays a central role. Such effects tightly couple the equations that govern the dynamics of fluids and, therefore, make the solution of each problem a case in itself.

### 2.1. First Generation of Drug-Eluting Stents

The first two original drug-eluting stents, called Cypher and Taxus, were approved by the Food and Drug Administration (FDA) after scientific evidence reported efficacy in reducing restenosis compared to bare-metal stents [20,21,22,23,24,25]. They were conceived from the combination of a stainless steel scaffold and a permanent polymer coating with the characteristic of releasing either sirolimus in the case of the Cypher stent or paclitaxel for the Taxus stent. The choice of these two drugs was motivated by the fact that they had shown efficacy in preventing smooth muscle cell (SMCs) proliferation and migration [26,27], while neointimal atherosclerotic change (neo-atherosclerosis) after BMS implantation in patients who were managed with a BMS occurred beyond 5 years. However, the DES was associated with decreased endothelialization and retarded recovery, resulting in increased risk of late in-stent thrombosis. The pathophysiological process seems to be related to a drug-mediated (Cypher and Taxus) inhibition of endothelial cell (EC) proliferation; furthermore, local drugs have paracrine effects on SMCs and inflammatory cells [9,10,11,28,29,30]. The local inflammatory response of the coronary endothelium can be prolonged depending on the chemical characteristics of the medications used [31].

There is currently a substantial body of circumstantial evidence to support that both DESs and BMSs are affected by the neo-pathoanatomical process of neo-atherosclerosis. Indeed, nine years ago, a study compared the incidence of neo-atherosclerosis after the use of a DES or BMS from autopsy cases (*n* = 299) [32]. Of these, 197 were BMSs while 209 were DESs. Sirolimus-eluting stents (SESs) were implanted 103 times and paclitaxel-eluting stents (PESs) 106 times. The occurrence of neo-atherosclerosis was greater in DES than BMS injuries (31% vs. 16%, *p* < 0.001). The median stent duration with neo-atherosclerosis was reduced among patients with a DES compared to those with a BMS (DES, 420 days [interquartile range, IQR: 361–683 days]; BMS, 2160 days [IQR: 1800–2880 days], *p*< 0.001). Incidences of unstable lesions characterized as plaque ruptures or thin-cap fibroatheromas were higher in persons who were managed with a BMS (4% vs. 1%; *p* = 0.17), with relatively shorter implant durations in DES arms (1.5 +/− 0.4 years vs. 6.1 +/− 1.5 years). Neo-atherosclerosis was a frequent finding in patients treated with PCI and DES and it manifested earlier than in those in whom PCI was combined with the use of a BMS. Interestingly, the instability characteristics of neo-atherosclerosis were identified for both BMSs and DESs, but the DES was associated with shorter implantation duration. Therefore, the process of neo-atherosclerosis may be considered another supporting element to late thrombotic events.

The TYPHOON randomized clinical trial [33] (Trial to estimate the use of the cYPHer sirolimus-eluting coronary stent in acute myocardial infarction treated with ballOON angioplasty) supported those results. The four-year follow-up revealed that patients who underwent PCI using an SES have reduced target lesion revascularization which was significantly better compared to those who received a BMS (92.4% vs. 85.1%; *p* = 0.002). However, no survival difference (97.6% and 95.9%; *p* = 0.37), repeat myocardial infarction (94.8% and 95.6%; *p* = 0.85), or stent thrombosis (SES: 4.4%, BMS: 4.8%, *p* = 0.83) were noted when comparing the two groups.

In the past 10 years, two independent randomized clinical trials (RCTs) have compared DESs with BMSs in larger cohorts of patients with longer-term follow-up (Table 1). One RCT [24] compared a sirolimus-eluting stent with a BMS among 1058 patients with recent CAD diagnosis. Patients who underwent PCI with the use of s BMS had a rate of failure of 21.0% compared to 8.6% in patients who were managed with a sirolimus-eluting stent (*p* < 0.001). This reduction was extensively associated with a decreased frequency of repeated revascularization in the target lesion (16.6% in BMS vs. 4.1% in sirolimus group, *p* < 0.001). In the sirolimus-eluting stent group, the incidence of in-stent neointimal hyperplasia was also decreased. Patients with complex CAD managed with a sirolimus-eluting stent showed benefits and a reduced rate of restenosis and associated adverse clinical events. Another multicentric study included 66 institutions with 1156 patients over a follow-up of 5 years and reported a reduced 9-month rate of target lesion revascularization in patients who received a BMS compared to those who had paclitaxel-eluting stents (15.7% to 8.6%; *p* < 0.001) and target vessel revascularization (17.3% to 12.1%; *p* = 0.02). Cardiac death and myocardial infarction had similar occurrence in both groups (about 5.5% in both groups), as well as stent thrombosis (<1% in both groups). Patients with complex coronary lesions managed with paclitaxel-eluting stents showed effectively reduced clinical and angiographic restenosis compared to those treated with BMS.

In the Intracoronary Stenting and Angiographic Results: Drug Eluting Stents for In-Stent Restenosis 2 (ISAR Desire 2) RCT [34], 450 patients with a sirolimus-eluting stent (SES) who had restenosis and required reintervention with a repeat SES implantation (*n* = 225) (Cypher, Cordis, Miami Lakes, Florida) were compared with those who underwent paclitaxel-eluting stent implantation (PES *n* = 225) (Taxus, Boston Scientific, Natick, Massachusetts) (Table 1). In patients who had SES restenosis and received either repeat SES or PES, the degree of efficacy and safety was comparable. In all ISAR (ISAR Desire and Smart II) [35,36] RCTs, the use of a PES was not inferior to SES (Table 1).

Despite these studies revealing substantial differences in pharmacodynamic and kinetics when comparing paclitaxel and sirolimus, the action of paclitaxel through the arterial wall resulted in a marked accumulation of this drug in the adventitia of vessel walls rather than the media. With the use of a simulation model, evidence from several studies [6,31] showed that paclitaxel separated from tissue more slowly than sirolimus by approximately 20 times, thus favoring a more permanent residency in the arterial wall compared to sirolimus. Concerns about this kinetic feature of the drug have meant that the Taxus stent has the physical characteristics of conveying a relatively high dose of paclitaxel for a period of 30 days. Therefore, the tendency to accumulate with very high levels in the arterial wall triggered localized inflammation [15,37]. This aspect has been highlighted in several studies on the efficacy of first-generation DESs, explaining why paclitaxel-eluting stents were less effective than sirolimus-eluting stents [38,39]. This problem can be resolved by decreasing the paclitaxel loading concentration in the stents by intervening in two stages. In the first phase, a higher dose of the drug is released in a short time, followed by a second phase in which the release is slower over a period of years. This model can reduce the amount of drug accumulating in the arterial wall, preventing SMC hyperplasia with minimal effects on the healing process [15,40]. A balloon model for paclitaxel release angioplasty that features a large initial burst release was used. Although the use of paclitaxel-eluting balloons has reduced restenosis rates compared to traditional angioplasty balloons [35,41,42], the use of PSEs has largely been eliminated from the PCI armamentarium. A complete investigation of the drug release kinetics appears crucial to improve clinical outcomes. 

### 2.2. Second Generation of Drug-Eluting Stents

The long-term safety and efficacy of BMSs is a matter of debate due the higher risk of restenosis after implantation, strongly supported by the emergence of DESs in the PCI scenario. There is indisputable evidence from a network meta-analysis, reporting a median follow-up of nearly 4 years, that despite a demonstrable benefit of the first generation of drug-eluting stents, the second generation reduced the late safety issues that were evident with first-generation DESs. The use of the second generation of drug-eluting stents had a greater late safety and effectiveness performance compared to bare-metal stents.

Given the successes of the first generation of DESs, the second generation of drug-eluting stents heralded an improvement over the previous Taxus and Cypher models with a greater emphasis on drug release kinetics, more efficient geometry, and advances in the biocompatibility of the materials used. The PCI platform was enriched with a new addition: cobalt-chromium everolimus-eluting stents (CoCr-EESs) (Abbott Vascular, Santa Clara, CA, USA), platinum-chromium EESs (PtCr-EESs) (Boston Scientific, Massachusetts, US), phosphorylcholine-based zotarolimus-eluting stent (PC-ZES), Resolute ZES (Re-ZES) (Medtronic), and biolimus-eluting stent (BES) (BioMatrix, Biosensors, Newport Beach, CA, USA; and Nobori, Terumo Clinical Supply, Kakamigahara, Japan). The latter was widely investigated in several large RCTs [43,44,45,46,47] (Table 2). The changes that were made to these devices resulted in improved clinical results, especially with regards to safety, in patients who received the second generation of DES compared to its predecessors and BMSs; it is thereby considered the current gold standard with superior clinical results [48,49,50]. 

Evidence from two independent meta-analyses based on larger cohorts of patients with longer-term follow-ups strongly suggested that the use of second-generation DESs was beneficial. In the first analysis [51], a total of 52,158 patients were enrolled for randomization. During a median follow-up of 3.8 years, the authors reported a significant decrease in death, definite stent thrombosis (ST), and myocardial infarction with the use of cobalt-chromium everolimus-eluting stents (EESs) compared to the use of BMSs, paclitaxel-eluting stents (PESs), and sirolimus-eluting stents (SESs). Patients who were managed with an EES had less ST than those who received a biolimus-eluting stent (BES). In addition, the authors noted that late target vessel revascularization rates were reduced in all patients where the second-generation DESs cobalt-chromium EES, platinum-chromium EES, SES, and BES were used compared to patients receiving a BMS. The second-generation DES recipients mentioned above had lower target vessel revascularization rates than PES. In the median follow-up period of almost 4 years, DES treatment was found to be superior to BMS treatment. Among DESs, usage of second-generation devices substantially improved long-term safety and efficacy outcomes compared to use of first-generation devices (Table 2).

In another study, 117,762 patient-years of follow-up were evaluated and patients were recruited from 76 randomized clinical trials. The results focused on the efficacy of BMSs against each DES (sirolimus-eluting stent (SES), paclitaxel-eluting stent (PES), everolimus-eluting stent (EES), zotarolimus-eluting stent (ZES), and ZES-Resolute (ZES-R)) [52] (Table 1 and Table 2). The results showed that in patients with reduced long-term target vessel revascularization, a higher percentage of this occurred in BMS recipients than in those in whom DESs were used (39% vs. 61%). However, the authors noted that the magnitude varied depending on the type of DES implanted (EES > SES > SEZ-R > PES > SEZ > BMS), showing a > 42% probability that EESs had the lowest target vessel revascularization rate. After the use of SESs, ZES-Rs, and everolimus-eluting stents, the short-term results were similar to the long-term results, whereby these were the most effective. Among the second-generation DESs, the EES was the safest. Safety endpoints remained stable throughout the study period, and stent thrombosis occurrence was similar between DESs and BMSs. However, there was a reduction in myocardial infarction rates and in the incidence of stent thrombosis for all recipients of a DES, except for those who had PESs versus BMSs (EES vs. BMS: rate ratio, 0.51; 95% credibility interval, 0.35–0.73) (Table 1 and Table 2).

Despite the optimistic results of second-generation DESs, the evident improvements did not reduce the risk of delayed in-stent thrombosis [12], indicating that a different approach to the stent was needed in its design. Furthermore, the role of the second-generation drugs used in bioabsorbable polymer-coated DESs should be clarified [53]. Notably, with the exception of the PROTECT study, which compared the long-term outcomes of SES and PC-ZES usage [54,55], concerns related to the long-term safety and efficacy of second-generation DESs persist as they have not been evaluated or investigated by adequately powered studies. A network meta-analysis may be able to overcome this drawback and increase the strength of the studies to be taken into consideration for guidelines.

## 3. New Frontiers of Stenting

Despite the strong increase in the PCI and stenting procedure using the second generation of DES, thrombosis, and restenosis of stents remain the Achilles heel of the procedure. For this reason, research has shifted to other design approaches for the development of new stents. The use of heparin incorporated in the device, negating the prothrombotic components on the stent, has been a new direction for the prevention of thrombosis. The use of heparin impregnated on the surface of the stent can be a valid option to prevent restenosis of the device due to the reactive formation of thrombi on the metallic core. The percutaneous procedures can use a commercially available heparin releasing stent. This device known as Viabahn is made by assembling a nitinol core which is coated with ePTFE and non-mobilizable heparin. The Viabahn stent has been proven to have better patency rates in clinical trials than the bare-metal stent [56,57].

Patients who have a sensitivity to polymers can benefit from polymer-free drug-eluting stents. A number of devices have been used in which even the loading of drugs onto the metal surface can be challenging, with results that are effective after stent implantation. Carrie et al. [58] investigated the effectiveness of the Cre8 stent, in which amphilimus is integrated into reservoirs of nanoparticles arranged on the abluminal side of the stent. Urban et al. [59] created a BioFreedom stent in which biolimus adheres to a microstructured metal surface. Another innovative stent is in VESTA sync, which is combined with a microporous coating of hydroxyapatite [60,61]. 

In particular, three different large-scale RCTs (SORT OUT V, COMPARE II, and LEADERS) showed that stents eluting biolimus from a biodegradable polymer are a safe and effective alternative to sirolimus [45,47] or everolimus [46]. As for the stent that integrates the biodegradable polymer biolimus, it is evident that the optimal clinical results obtained by these more complex stent models are due to better optimization of the drug release kinetics, in addition to the material and mechanical properties. The advantage of these stents is that they have reservoirs that can be filled with drugs. They are progressively released through small perforations on the luminal side of the stent, allowing a more sustained and targeted drug administration [62]. In particular, cobalt-chromium stents have been combined with polymerized paclitaxel or everolimus [46,63].

The innovative design with dynamic and mechanical features of the layer-by-layer assembly system to coat the stents proved effective. Chitosan and hyaluronic acid are generally chosen as materials and enhanced with growth factors or heparin to customize drug release kinetics [64,65,66,67,68,69]. For example, released coatings with a combination of sirolimus and heparin have been shown to have a favorable action in preventing restenosis and thrombosis, respectively [66].

The action of heparin is also manifested on growth factors due to their high affinity, which are sequestered on the heparin surface. Liu et al. [69] showed that heparin was rendered inactive on the stent surface using an avidin-biotin system, and thus, CD34 and VEGF are embedded to heparin to accelerate endothelialization. Our group achieved the same effect with poly-L-lactide (PLLA) [64,70,71], and these studies showed promising results in vitro and in vivo.

## 4. Bioresorbable Vascular Scaffolds

The use of a bioresorbable vascular stent (BDES or BVS) in clinical practice has been suggested to overcome DES limitations such as in-stent restenosis. Bioresorbable DESs or vascular scaffolds (BVSs) were initially designed from metallic or polymeric compounds (Table 3).

Recently, many companies have been researching new stent designs in response to concerns about thrombosis caused by the long-term implantation of second-generation DESs. In January 2011, Abbott announced the European approval of ABSORB, the world’s first bioresorbable vascular stent (BDES) for coronary artery disease. Absorb™ was approved by the FDA in 2016 but was later removed from the global market. Nevertheless, there has been continual development in this market, with several new innovations awaiting approval or in clinical trials. The BDES consists of the combination poly-D, L-lactide (PDLLA), more commonly known as PLLA, with everolimus or novolimus. The most widely commercially used BDESs are the ABSORB stent and the DESolve stent, which are a combination of a dimeric shape of PLLA and everolimus (ABSORB) or novolimus (DESolve). Recently, another BDES, magnesium-based scaffolds (DREAMS 2G), functioning as degradable metals, was approved for clinical use. The only commercially available DREAMS 2G BDES consists of a magnesium alloy with a sirolimus-loaded PLA coating, approximately 95% of which resorbs within one year of implantation. During the year of bio-reabsorption, the magnesium compound degrades. The last stage of the transformation is amorphous calcium phosphate, which remains within the tissue. Haude et al. [72], in a randomized clinical trial, showed that the DREAMS 2G BDES demonstrated similar results to the use of other commercially available polymeric bioresorbable vascular scaffolds, but tailored studies with direct comparisons are awaited.

Reabsorption time varies between 1 (DESolve) and 3 (Absorb) years, but after the external material is reabsorbed, the coronary artery does not contain persistent structures, which can be daunting if subsequent coronary surgery is required. In fact, the surgeon performing the CABG surgery can intervene on small vessels that are free from the free presence of the metal component of the stent because the BDES is completely degrading. Hence, surgeons can operate more comfortably and can alleviate many of the negative effects seen with metal–polymer coatings. Another concern is related to the fact that the stents are often inserted in the part of the coronary artery that has the best caliber, thus forcing the surgeon to perform the bypass in the most distal part of the vessel that has a smaller caliber.

From a pathophysiological point of view, Serruys et al. [73] noted that the use of a BDES determines the return of the physiological function of the vessel. Non-degradable stents favor a permanent focal decrease in vascular compliance, leading to a mismatch of regional compliance which is a contributing factor to restenosis [74,75]. While awaiting the outcome of the ABSORB trial at 5 years, there has been a substantial body of circumstantial evidence to support the use of BDESs, which offer an additional benefit on restenosis of the stent, with a similar risk of death when compared to the second generation of DESs [73,76,77,78,79].

The randomized clinical trial ABSORB III, which enrolled 2084 patients, compared the use of the BDES Absorb (*n* = 1322) versus the everolimus-eluting Xience DES (*n* = 686) and was the pilot study. The results of the ABSORB trials showed good performance compared to everolimus DESs. However, slightly poorer outcomes impairing any long-term benefits were recognized. 

As for bioresorbable stents, their expected benefits would be noted when the stent dissolves, generally after three years [80]. However, these benefits were not shown in the ABSORB III trial, and the device carried several disadvantages, including demonstrable poorer outcomes compared to DESs [80] in terms of target lesion failure. In addition, the results showed that stent thrombosis of the target lesion and MI were higher with this device [80,81]. 

These results were confirmed in a recent meta-analysis involving 3384 patients. In a 5-year follow-up period, patients who received BVSs compared to those who underwent the use of EESs were associated with higher rates of target lesion failure (TLF) (14.9% vs. 11.6%; HR, 1.26; 95% CI, 1.03–1.54; *p*  =  0.03) and device thrombosis (2.5% vs. 0.8%; HR, 2.87; 95% CI, 1.46–5.65; *p*  =  0.002). Target lesion failure occurred in 11.6% of BDES patients vs. 7.9% of EES patients who received an EES between 0 and 3 years (HR, 1.42; 95% CI, 1.12–1.80), and 4.3% of BDES-treated patients vs. 4.5% of EES-treated patients between 3 and 5 years (HR, 0.92; 95% CI, 0.64–1.31) (*p* for interaction  =  0.046). Device thrombosis was observed in 2.4% of recipients of a BDES vs. 0.6% of patients who had EESs between 0 and 3 years (HR, 3.86; 95% CI, 1.75–8.50) and 0.1% of BDES-treated patients vs. 0.3% of patients who underwent the procedure with the use of EESs between 3 and 5 years (HR, 0.44; 95% CI, 0.07–2.70) (*p*  = 0.03) [81]. The major concern with Absorb/BDES is that the risk/benefit ratio is optimal at 3 years, with an increased risk of complications after this period. 

Further investigations are required to clarify the concerns related to very late scaffold thrombosis that may occur at advanced stages of scaffold resorption. Potential mechanisms specific for very late scaffold thrombosis include scaffold discontinuity and restenosis during the resorption process, which may be delayed in humans; this suggests an extended period of vulnerability for thrombotic events [8,82]. Although the remodeling capacity of the endothelium of vessel walls using two types of resorbable material is enhanced, BVSs demonstrate very intense cell proliferative activity both at the level of CD31 cells that differentiate towards endothelial-like morphology and towards cells that produce fibronectin with the use of a BVS [64,83,84]. The BVS showed higher production of new extracellular matrix that was mainly characterized by a higher content of elastin fibers in the vessel wall and a more compact organization of collagen fibers in the elastic zone of the vessel [64,83,84]. Interestingly, we demonstrated overexpression of the metalloprotease MMP-9, which indicates an ongoing matrix remodeling process [35,83,84]. In parallel, cell proliferation was found to be increased in recipients of BVS as testified by the significantly higher percentage of ki67-positive cells (26.89% 68.4% in BVS vs. 51.55% 69.7% in non-BVS *p* < 0.05). These findings were coupled with a significant reduction in apoptosis in BVS recipients, supporting the idea of an active remodeling process in these recipients (47.8% +/− 7.2% in non-BVS vs. 17.5% +/− 5.1% in BVS, *p* < 0.05) [85,86]. 

A recent paper compared polymer-free vs. polymer-coated DESs in a meta-analysis of 16 RCTs [87]. After a median follow up of 2 years, polymer-free DESs might be associated to reduced mortality compared to polymer-coated DESs (HR 0.82, 95% CI 0.68–0.99, *p* = 0.03), but no differences were observed in other significant endpoints (major ischemic events, cardiovascular death, myocardial infarction, or TLR). However, the authors point out that particular categories of risk (increased risk of bleeding events or recent MI) should be adequately investigated in future clinical trials and in future stent design [87].

Considering secondary evidence about the comparison between a drug-coated balloon and a DES, two recent meta-analyses focused on small coronary arteries [88] and large vessels [89]. In patients with narrowed arteries [88], balloons reduced the risk of coronary thrombosis (OR 0.12; 95% CI 0.01–0.94; *p* = 0.04) at the expense of a poorer angiographic result in terms of luminal diameter and percentage diameter stenosis, while TVR and restenosis rates were comparable. In patients with large vessels [89], the balloons seem non-inferior to DESs after 6–9 months after PCI, with no differences in late lumen loss (SMD, −0.07; *p* = 0.548) and TLR (RR, 1.17; *p* = 0.746). Those results highlight the impact of the diameter of native coronary arteries in the results of percutaneous procedures and might suggest a tailored approach for current clinical use and future studies.

## 5. Drug Delivery Options for Cardiovascular Interventions: How and When

**Figure 1:**
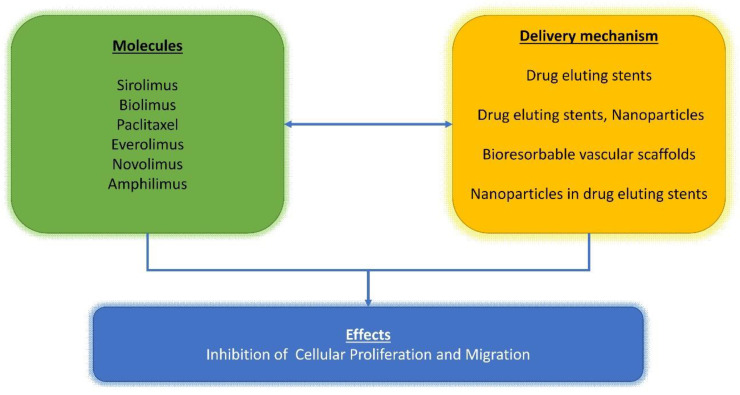
Schematic representation of different pathways described in the text. Effect of sirolimus and similar compounds (biolimus, paclitaxel, everolimus, novolimus, and amphilimus) (green box) and their delivery mechanisms (yellow box) and downstream effects (blue box).

**Figure 2:**
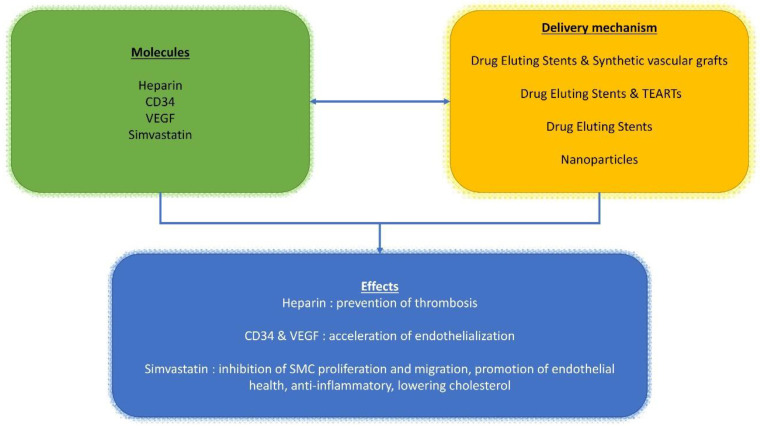
Schematic representation of different pathways described in the text. Effect of heparin, CD34, VEGF and simvastatin (green box) and their delivery mechanisms (yellow box) and downstream effects (blue box).

**Figure 3:**
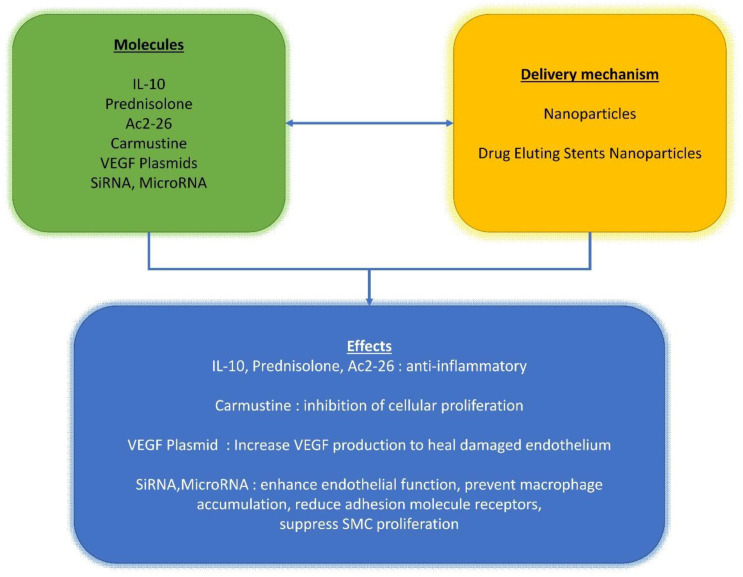
Schematic representation of different pathways described in the text. Effects of IL-10, Prednisolone, Ac2-26, carmustine, VEGF plasmids, siRNA, and microRNA (green box) and their delivery mechanisms (yellow box) and downstream effects (blue box).

The complexity of this topic is compounded with researchers studying the effects of combining drugs or growth factors into biomaterials used as prostheses or remodeling patches (Figure 1, Figure 2 and Figure 3). The most common is the use of molecules such as heparin that can be integrated on the material surface. This approach is often used to stimulate cell proliferation such as for its use in the electrospun poly-L-lactide (PLLA) tubular scaffold [65,71,90] or when used to reduce the risk of thrombotic complication in implanted synthetic vascular grafts [91]. The final purpose of the use of heparin is to support cell differentiation and realize a drug delivery device to anticipate graft thrombosis of engineered tissues applied to the arteries (TEARTs) for vascular intervention. In addition, Jeon reported the peculiar activity of heparin-coated surfaces that can be used to bind growth factors [92]. The authors achieved an improvement in ectopic bone formation by bone-morphogenetic-protein-2 released from a heparin-containing poly-(L-lactic-co-glycolic acid) scaffold. We conceived a granulocyte colony-stimulating factor (GCSF)-releasing polymeric scaffold in poly-L-lactide (PLLA) electrospun fibers in which we cultured skeletal myoblasts to obtain a tissue-engineered cardiac graft (TECG) [70], which was used as a ventricular patch in an animal model of chronic myocardial infarction. We employed a GCSF which is known to mobilize endogenous bone marrow (BM)-derived cells [93].

Another system that provides a valid alternative for therapeutic use is encapsulating pharmacologically active substances within biomaterials during manufacture. A hybrid technique associating electrospinning and bioprinting was used to fabricate a bioresorbable scaffold for vascular tissue engineering, with a single-layer helical poly-e-caprolactame (PCL) coil [64,71,90,94,95]. The vascular scaffold was bioprinted on the external surface to reinforce a heparin-releasing PLLA tubular electrospun scaffold [96,97,98]. Zhang also described a similar procedure of electrospinning emulsion [99].

Furthermore, mechanical properties appear to be crucial for tissue engineering. A reactive electrospinning approach might be helpful in attaining the required mechanical properties as it helps to safely and successfully upload different therapeutic hormones and drug moieties actively and stably [100,101].

An alternative approach is to insert the molecules via diffusion into the materials after fabrication. The material is immersed in a bioactive factor solution. Several studies reported how growth factors can spread in gelatin scaffolding and electrostatically bind to gelatin. This mechanism is based on the proteolytic degradation of gelatin. In fact, the growth factor is released by dissociation and diffusion when the proteolysis reaction occurs [102,103]. The kinetics of the materials used is a fundamental criterion to establish the efficacy of the drugs employed for bioptic action.

Biomaterials must fulfill some basic requirements for use as stent coatings or scaffolds such as non-toxicity, hemocompatibility, and the capability of supporting cell growth and vitality. Moreover, it is also important that the same materials promote antithrombotic and anti-inflammatory responses while accelerating endothelial growth and regeneration. In designing a “new” device, all of these goals are achieved by combining not only materials and drugs but also a suitable manufacturing process. When thinking about a polymer carrier, it is important that the mechanical properties as well as the hydrophilicity of the material are optimized to adapt the biomaterials to the physiological needs.

Polyesters and poly-anhydrides are common bio-absorbable polymers used as coating or scaffolds in coronary stents. The most common polyester is PLA, which degrades in 2–3 years. Three main distinct forms of PLA exist: (i) poly-L-lactide (PLLA), (ii) Poly-D-lactide (PDLA), and (iii) poly (DL-lactide) (PDLLA), i.e., a co-polymer of the previous two. The two main homo-polymers (PLLA and PDLA) are generally in a semi-crystalline form, while the latter, due to the lack of tacticity, is an amorphous one. Another polyester often used is poly(caprolactone), PCL. Compared with PLA, PCL has a shorter degradation time due to its lower crystallinity, which, in turn, confers the polymer a higher flexibility. Another biodegradable polymer employed in coronary implants is Poly (anhydride ester) salicylic acid (IDEAL). The mechanical properties of each polymer type vary according to the composition, molecular weight, copolymer additives, and, last but not least, the degree of crystallinity. The latter property is also adversely responsible for the degree of water absorption. Indeed, it is known that water can be uptaken only in the amorphous region of the polymer, while the crystalline ones are essentially impervious. A new family of Poly(decanediol-co-tricarballylate) polyesters has been recently and successfully used for tissue engineering of cardiac tissues using a photoreactive electrospinning approach [104]. This newly synthesized fibrous scaffold has been investigated in early studies, with promising results in view of its features that allow to withstand cardiac systole and diastole [104].

Sometimes, the mechanical features of a polymer should be adapted to fulfill project requirements. In these cases, chemical modification or polymer blending are the chosen strategies. However, such modifications also have an effect on degradation rates such as degradation which occurs through hydrolysis of the polymer chains.

## 6. Future Direction for the Stent Design

The success of stents is strongly focused on the kinetics of the polymers integrated into the device and their behavior, evaluated by in vivo tests (Figure 1, Figure 2 and Figure 3). Several iterative design improvements were required collaboratively between cardiologists, heart surgeons, histochemists, and mathematicians to achieve the desired result [84,85,86,105,106].

Mathematical models should be used to evaluate the kinetics of drug delivery. Simulations may produce stents that convey drug therapies at doses that impede the proliferation of smooth muscle cells and anticipate restenosis without negative effects on endothelial cells.

Many new molecules have appeared on the scene for possible use and promise to improve future drug-eluting stent models. Among these, very promising and under current investigation are the gene-eluting stents that can provide small interfering ribonucleic acid with siRNA release [68,107,108]. In this way, the stents that make use of embedded siRNA (RNA molecules impeding target gene expression) have as their objective a modulating effect on the receptors of the adhesion molecules to reduce thrombosis and inflammation [68] or to suppress the proliferation of SMCs, useful for preventing restenosis [85]. In particular, promising results were obtained using Akt1 siRNA nanoparticles (ASN) released from a stent surface coated with hyaluronic acid (HA). It was shown that this combination specifically suppressed the pro-proliferative protein Akt1 in smooth muscle cells (SMCs), avoiding restenosis. The therapeutic effects could be rapidly translated from the animal model to humans to concretely relieve the effects of in-stent restenosis [86]. In this direction, undertaking a histochemical analysis based on the use of anti-CD31 antibodies and anti-Ki67 antibodies [79] may help evaluate the re-endothelization process after implantation of an ASN-immobilized stent [86,109,110].

Evidence has shown that gene-eluting stents can adapt the local microenvironment to reduce hyperplasia and intimal thrombosis [111,112]. Future directions should be focused on developing newer stent materials and therapies available with tailored dosing kinetics. This evolution in personalization can determine the selection of the stent, which can be adapted to the needs of a single patient, offering the most advantageous dosage compared to one or more specific therapies for long-term patients for a precise duration.

Besides the applications in interventional cardiology, bioresorbable scaffolds are a hotly contested topic even in cardiac surgery. Engineered tissues applied to the arteries (TEARTs) and left ventricle have been recently regarded as substitutes to synthetic grafts for CABG, reconstruction of the left ventricle, and arterial procedures. The advantage of TEARTs is the more physiologic structure compared to synthetic materials. In this scenario, TEARTs resorption can facilitate remodeling of the extracellular matrix and cells of the vascular wall, leading to a vascular neo-structure with selective histochemical modifications.

## 7. Conclusions

This review summarizes the available evidence about the use of bioactive polymers for intervention and tissue engineering, with specific regards to cardiovascular disease. The widespread use of different polymers in coronary stenting and the multiple clinical implications that can be hypothesized, starting from basic science studies, should be carefully investigated in the cardiologic scenario. It appears crucial to summarize all the recent evidence on this topic with the aim of portraying the literary landscape for future tailored studies.

When an implanted device includes an active ingredient, its release into vessel walls and into the blood is strongly influenced by the hemodynamics around the device and the kinetics of release. The whole scenario is then challenging and fascinating, as it involves mechanics, fluid dynamics, and mass transfer processes, but a holistic approach is required to find the right solution to improve outcomes of stent and cardiac grafts. At present, newer-generation DESs have significantly reduced the burden of atherosclerotic coronary artery disease over the years. Bioresorbable scaffolds and balloons represent promising techniques that might be extremely helpful in particular subset of patients, such as those with diffusely diseased coronary arteries of previous stent failure.

## Figures and Tables

**Table 1:** Summary of Contemporary Series Comparing BMS and DES. Abbreviation. BMS = bare-metal stent; DES = drug-eluting stent; MI = myocardial infarction; PES = paclitaxel-eluting stent; RCT = randomized clinical trial; ST= stent thrombosis; SES = sirolimus-eluting stent; *TLF = target lesion failure; TLR = target lesion revascularization; TVR= target vessel revascularization. *TVF = target vessel failure, defined as cardiac death, target vessel MI, or TVR. **† References are in Supplementary Table S1**.

Author/Year † Ref	Type of Study/Randomization	Treatment Total Number	Maximum Follow-Up (yrs)	Stent Compared /*n* Implanted			Main Finding
**Valgimigli, 2013 [1]** ***Int. J. Cardiol.***	RCT 1:1 Multi-center	744	3	BMS 372	SES 372		Higher TVR failure based on death, MI, and clinically for BMS. SES was superior to BMS,
**Sinning, 2012 [2]** ***Am. Heart J.***	RCT 1:1 Multi-center	200	5	BMS 102	SES 98		Higher late luminal loss for BMS. SES was superior to BMS,
**Spaulding, 2011 [3]** ***JACC Cardiovasc. Interv.***	RCT 1:1 Multi-center	712	4	BMS 355	SES 357		Higher TVF for BMS. SES was superior to BMS.
**Mehilli, 2010 [4]** ***J. Am. Coll. Cardiol.***	RCT 1:1 Two centers	450	5	SES 250	PES 250		Higher late luminal loss for PES. SES not proved superior.
**Atary, 2010 [5]** ***AJC***	RCT 1:1 Single-center	310	5	BMS 152	SES 158		Higher late luminal loss in the coronary segment for BMS. SES was superior to BMS.
**Di Lorenzo, 2009 [6]** ***JACC Cardiovasc. Interv.***	RCT 1:1:1 Single-center	270	4	BMS 90	PES 90	SES 90	Higher TLR for BMS. PES and SES were superior to BMS.
**Mehran, 2008 [7]** ***Am. Heart J.***	RCT 3:1 Multi-center	3006	3	BMS 2257	PES 749		Higher TLR for BMS. No difference for death, MI, stroke, or ST. PES was superior to BMS for TLR and not inferior for clinical outcomes.
**Lee, 2008 [8]** ***Catheter Cardiovasc Interv.***	RCT 1:1 Multi-center	308	3	SES 154	PES 154		No difference between SES and PES for death, MI, ST, and *TLF, defined as cardiac death or target vessel MI. SES not proved superior.
**Menichelli, 2007 [9]** ***JACC***	RCT 1:1 Single-center	320	5	BMS 160	SES 160		Higher binary restenosis for BMS. SES was superior to BMS.
**Mehilli, 2006 [10]** ***Eur. Heart J.***	RCT 1:1 Two centers	360	5	SES 180	PES 180		Higher in-stent late luminal loss for PES. PES was inferior to SES.
**Suttorp, 2006 [11]** ***Circulation***	RCT 1:1 Two centers	200	3	BMS 100	SES 100		Higher grade of angiographic in-segment restenosis for BMS. SES was superior to BMS.
**Thuesen, 2006 [12]** ***Am. Heart J.***	RCT 1:1 Multi-center	322	3	BMS 159	SES 163		Inferior minimal lumen diameter for BMS. SES was superior to BMS.
**Valgimigli, 2005 [13]** ***JAMA***	RCT 1:1 Two centers	175	5	BMS 87	SES 88		Higher death, MI, stroke, and binary restenosis for BMS. SES was superior to BMS.
**Windecker, 2005 [14]** ***NEJM***	RCT 1:1 Single-center	1012	5	SES 503	PES 509		No difference between SES and PES for cardiac death, MI, TLR. SES not proved superior.
**Dibra, 2005 [15]** ***NEJM***	RCT 1:1 Two centers	250	5	SES 125	PES 125		Higher late luminal loss for PES. SES was superior to PES.
**Goy, 2005 [16]** ***J. Am. Coll. Cardiol*.**	RCT 1:1 Single-center	202	3	SES 102	PES 100		No difference between SES and PES for cardiac death, MI, and TLR. SES not proved superior.
**Holmes, 2004 [17]** ***Circulation***	RCT 1:1 Multi-center	1058	4	BMS 533	SES 525		Higher *TVF or *TVR for BMS. SES was superior to BMS.
**Stone, 2004 [18]** ***Circulation***	RCT 1:1 Multi-center	1314	5	BMS 662	PES 652		Higher TVR failure based on ischemia for BMS. PES was superior to BMS.
**Morice, 2002 [19]** ***NEJM***	RCT 1:1 Multi-center	238	4	BMS 120	SES 118		Higher in-stent late luminal loss for BMS. SES was superior to BMS.

**Table 2:** Summary of contemporary series comparing second-generation DESs. Abbreviations. BP-BES = biodegradable polymer biolimus-eluting stent; C-SES = Cypher sirolimus-eluting stent; CoCr-EES = cobalt-chromium everolimus-eluting stent; PC-ZES = phosphorylcholine-based zotarolimus-eluting stent; PtCr-EES = platinum-chromium everolimus-eluting stent; E-ZES = Endeavor zotarolimus-eluting stent. Other abbreviations are as in Table 1. **† References are in Supplementary Table S2.**

Author/Year † Ref	Type of Study/Randomization	Treatment Total Number	Maximum Follow-Up (yrs)	Stent Compared/*n* Implanted		Main Finding
**Jakobsen, 2017 [1]** ***EuroIntervention***	RCT 1:1 Multi-center	2468	3	BP-BES 1229	SES 1239	No difference for cardiac death, MI, definite ST, and clinically based on TVR. Non-inferiority for BP-BES has not been demonstrated,
**Raungaard, 2015 [2]** ***Lancet***	RCT 1:1 Multi-center	2999	5	BP-BES 1497	PC-ZES 1502	No difference for cardiac death and MI. PC-ZES was not inferior to BP-BES.
**Smits, 2015 [3]** ***JACC Cardiovasc. Interv.***	RCT 1:1 Single-center	1800	5	CoCr-EES 897	PES 903	Higher death, MI, and TVR for PES. CoCr-EES was superior to PES.
**Iqbal, 2015 [4]** ***Circ Cardiovasc Interv.***	RCT 1:1 Multi-center	2292	4	CoCr-EE 1152	Re-ZES 1140	No difference for TLF. Re-ZES was not inferior to CoCr-EES.
**Natsuaki, 2015 [5]** ***Catheter Cardiovasc Interv.***	RCT 3:2 Multi-center	326	3	BP-BES 194	SES 132	No difference for TVF. BP-BES was not inferior to SES.
**Maeng, 2014 [6]** ***Lancet***	RCT 1:1 Multi-center	2332	5	SES 1170	PC-ZES 1162	Higher cardiac death, MI, and TVR for PC-ZES. SES was superior to PC-ZES.
**Di Lorenzo, 2014 [7]** ***JACC Cardiovasc. Interv.***	RCT 1:1 Single-center	500	3	EES 250	SES 250	No difference for cardiac death and reinfarction. EES similar efficacy as SES. EES proved significant reduction in ST.
**Serruys, 2013 [8]** ***JACC Cardiovasc. Interv.***	RCT 1:1 Multi-center	1707	4	BP-BES 875	SES 875	No difference for cardiac death, MI, and TVR. BP-BES was not inferior to SES.
**Jensen, 2012 [9]** ***Circulation***	RCT 1:1 Multi-center	2774	5	CoCr-EES 1390	SES 1384	No difference for cardiac death, MI, definite ST, and TVR. CoCr-EES was not inferior to SES.
**Kandzari, 2011 [10]** ***JACC Cardiovasc. Interv.***	RCT 1:3 Multi-center	436	5	SES 113	PC-ZES 323	Higher grade of late lumen loss for PC-ZES. PC-ZES was inferior to SES.
**Stone, 2011 [11]** ***J. Am. Coll. Cardiol.***	RCT 1:1 Multi-center	1530	3	PtCr-EES 768	CoCr-EES 762	No difference for TLF. PtCr-EES was not inferior to CoCr-EES.
**Leon, 2010 [12]** ***J. Am. Coll. Cardiol.***	RCT 1:1 Multi-center	1548	3	PES 775	PC-ZES 773	No difference for TVF. PES was not inferior to PC-ZES.
**Kereiakes, 2010 [13]** ***JACC Cardiovasc. Interv.***	RCT 2:1 Multi-center	1002	5	CoCr-EES 699	PSE 333	Higher-grade in-segment late luminal loss and higher TVR for PES. CoCr-EES was superior to PES.
**Byrne, 2009 [14]** ***Eur. Heart J.***	RCT 1:1 Two centers	1304	3	CoCr-EES 652	SES 652	No difference for cardiac death, MI, and TLR. CoCr-EES was not inferior to SES.
**Nicolsky, 2009 [15]** ***Am. Heart J.***	RCT 2:1 Multi-center	3687	3	CoCr-EES 2458	PES 1229	Higher TLF or TLR defined as cardiac death or target vessel MI for PES. CoCr EES was superior to PES.
**Camenzind, 2009 [16]** ***Am. Heart J.***	RCT 1:1 Multi-center	8791	4	C-SES 4352	E-ZES 4357	No difference for ST. E-ZES was not superior to C-SES.
**Garg, 2009 [17]** ***JACC Cardiovasc. Interv.***	RCT 3:1 Multi-center	300	3	CoCr-EES 233	PSE 77	No difference for in-stent late luminal loss. CoCr-EES was not inferior to PES.
**Fajadet, 2006 [18]** ***Circulation***	RCT 1:1 Multi-center	1197	5	PC-ZES 598	BMS 599	Higher TVF for BMS. PC-ZES was superior to BMS.
**Chevalier, 2006 [19]** ***EuroIntervention***	RCT 1:2 Multi-center	120	5	BP-BES 35	PES 85	No difference in-stent late luminal loss. BP-BES was not inferior to PES.
**Smits, 2005 [20]** ***Lancet***	RCT 1:2 Multi-center	2707	3	CoCr-EES 912	BP-BES 1795	No difference for cardiac death, non-fatal MI, and TVR. BES was not inferior to CoCr-EES.

**Table 3:** Summary of the polymers currently used in stents and balloons.

Commercial Name	Compound
**PES**	Paclitaxel
**BES**	Biolimus
**BP-BES**	Biodegradable polymer biolimus
**SES**	Sirolimus
**C-SES**	Cypher sirolimus
**EES**	Everolimus
**CoCr-EES**	Cobalt-chromium everolimus
**PtCr-EES**	Platinum-chromium everolimus
**Re-ZES**	Resolute zotarolimus
**E-ZES**	Endeavor zotarolimus
**PC-ZES**	Phosphorylcholine zotarolimus
**SPC-ZES**	Phosphorylcholine polymer-based zotarolimus

## Supplementary Materials

The following are available online at https://www.mdpi.com/2073-4360/13/3/446/s1.
