# Supplementary material for: The Use of Bioactive Polymers for Intervention and Tissue Engineering: The New Frontier for Cardiovascular Therapy
Source: Polymers (Basel). 2021 Jan 30;13(3):446. doi: 10.3390/polym13030446 (PMC7866823; doi:10.3390/polym13030446)
Supplement: Supplementary file 1 [file polymers-13-00446-s001.pdf]

polymers

MDPI

Supplementary Reference Table S1

1. Valgimigli M, Campo G, Gambetti S, Bolognese L, Ribichini F, Colangelo S, de Cesare N, Rodriguez AE, Russo F, Moreno R, Piva T, Sheiban I, Penzo C, Prati F, Nazzaro MS, Díaz Fernández JF, Vassanelli C, Parrinello G, Ferrari R. MULTIcentre evaluation of Single high-dose bolus TiRofiban versus Abciximab with sirolimus eluting sTEnt or Bare Metal Stent in Acute Myocardial Infarction studY Investigators. Three-year follow-up of the MULTIcentre evaluation of Single high-dose Bolus TiRofiban versus Abciximab with Sirolimus-eluting STEnt or Bare-Metal Stent in Acute Myocardial Infarction StudY (MULTISTRATEGY). Int J Cardiol. 2013 Apr 30;165(1):134-41. doi: 10.1016/j.ijcard.2011.07.106. Epub 2011 Aug 24.
2. Sinning JM, Baumgart D, Werner N, Klauss V, Baer FM, Hartmann F, Drexler H, Motz W, Klues H, Voelker W, Pfannebecker T, Stoll HP, Nickenig G; SCORPIUS Study. Five-year results of the Multicenter Randomized Controlled Open-Label Study of the CYPHER Sirolimus-Eluting Stent in the Treatment of Diabetic Patients with De Novo Native Coronary Artery Lesions (SCORPIUS) study: a German multicenter investigation on the effectiveness of sirolimus-eluting stents in diabetic patients. Am Heart J. 2012 Mar ;163(3):446-53, 453.e1. doi: 10.1016/j.ahj.2011.12.010
3. Spaulding C, Teiger E, Commeau P, Varenne O, Bramucci E, Slama M, Beatt K, Tirouvanziam A, Polonski L, Stella PR, Clugston R, Fajadet J, de Boisgelin X, Bode C, Carrié D, Erglis A, Merkely B, Hosten S, Cebrian A, Wang P, Stoll HP, Henry PFour-year follow-up of TYPHOON (trial to assess the use of the CYPHer sirolimus-eluting coronary stent in acute myocardial infarction treated with BallOON angioplasty).. JACC Cardiovasc Interv. 2011 Jan ;4(1) :14-23. doi : 10.1016/j.jcin.2010.10.007
4. Mehilli J, Byrne RA, Tiroch K, Pinieck S, Schulz S, Kufner S, Massberg S, Laugwitz KL, Schömig A, Kastrati A ; ISAR-DESIRE 2 Investigators.Randomized trial of paclitaxel- versus sirolimus-eluting stents for treatment of coronary restenosis in sirolimus-eluting stents : the ISAR-DESIRE 2 (Intracoronary Stenting and Angiographic Results : Drug Eluting Stents for In-Stent Restenosis 2) study.

   J Am Coll Cardiol. 2010 Jun 15 ;55(24) :2710-6. doi: 10.1016/j.jacc.2010.02.009. Epub 2010 Mar 11
5. Atary JZ, van der Hoeven BL, Liem SS, Jukema JW, van der Bom JG, Atsma DE, Bootsma M, Zeppenfeld K, van der Wall EE, Schalij MJ. Three-year outcome of sirolimus-eluting versus bare-metal stents for the treatment of ST-segment elevation myocardial infarction (from the MISSION ! Intervention Study). Am J Cardiol. 2010 Jul 1 ;106(1) :4-12. doi : 10.1016/j.amjcard.2010.02.005. Epub 2010 May 13.

6. Di Lorenzo E, De Luca G, Sauro R, Varricchio A, Capasso M, Lanzillo T, Manganelli F, Mariello C, Siano F, Pagliuca MR, Stanco G, Rosato G.. The PASEO (PaclitAxel or Sirolimus-Eluting Stent Versus Bare Metal Stent in Primary Angioplasty) Randomized Trial. JACC Cardiovasc Interv. 2009 Jun ;2(6) :515-23. doi : 10.1016/j.jcin.2009.03.012
7. Mehran R, Brodie B, Cox DA, Grines CL, Rutherford B, Bhatt DL, Dangas G, Feit F, Ohman EM, Parise H, Fahy M, Lansky AJ, Stone GW.The Harmonizing Outcomes with RevasculariZatiON and Stents in Acute Myocardial Infarction (HORIZONS-AMI) Trial: study design and rationale. Am Heart J. 2008 Jul ;156(1) :44-56. doi : 10.1016/j.ahj.2008.02.008
8. Lee JH, Kim HS, Lee SW, Park JH, Choi SW, Jeong JO, Cho Y, Lee N, Rhee KS, Ko JK, Seong IW. Prospective randomized comparison of sirolimus- versus paclitaxel-eluting stents for the treatment of acute ST-elevation myocardial infarction : pROSIT trial. Catheter Cardiovasc Interv. 2008 Jul 1 ;72(1) :25-32. doi : 10.1002/ccd.2151
9. Menichelli M, Parma A, Pucci E, Fiorilli R, De Felice F, Nazzaro M, Giulivi A, Alborino D, Azzellino A, Violini R. Randomized trial of Sirolimus-Eluting Stent Versus Bare-Metal Stent in Acute Myocardial Infarction (SESAMI). J Am Coll Cardiol. 2007 May 15;49(19):1924-30. doi: 10.1016/j.jacc.2007.01.081. Epub 2007 Apr 30
10. Mehilli J, Dibra A, Kastrati A, Pache J, Dirschinger J, Schömig A; Intracoronary Drug-Eluting Stenting to Abrogate Restenosis in Small Arteries (ISAR-SMART 3) Study Investigators. Randomized trial of paclitaxel- and sirolimus-eluting stents in small coronary vessels. Eur Heart J. 2006 Feb ;27(3) :260-6. doi : 10.1093/eurheartj/ehi721. Epub 2006 Jan 9
11. Suttorp MJ, Laarman GJ, Rahel BM, Kelder JC, Bosschaert MA, Kiemeneij F, Ten Berg JM, Bal ET, Rensing BJ, Eefting FD, Mast EG. Primary Stenting of Totally Occluded Native Coronary Arteries II (PRISON II) : a randomized comparison of bare metal stent implantation with sirolimus-eluting stent implantation for the treatment of total coronary occlusions. Circulation. 2006 Aug 29 ;114(9) :921-8. doi : 10.1161/CIRCULATIONAHA.106.613588. Epub 2006 Aug 14
12. Thuesen L, Kelbaek H, Kløvgaard L, Helqvist S, Jørgensen E, Aljabbari S, Krusell LR, Jensen GV, Bøtker HE, Saunamäki K, Lassen JF, van Weert A ; SCANDSTENT Investigators Comparison of sirolimus-eluting and bare metal stents in coronary bifurcation lesions : subgroup analysis of the Stenting Coronary Arteries in Non-Stress/Benestent Disease Trial (SCANDSTENT).Am Heart J. 2006 Dec;152(6):1140-5. doi : 10.1016/j.ahj.2006.06.035

13. Valgimigli M, Percoco G, Malagutti P, Campo G, Ferrari F, Barbieri D, Cicchitelli G, McFadden EP, Merlini F, Ansani L, Guardigli G, Bettini A, Parrinello G, Boersma E, Ferrari R ; STRATEGY InvestigatorsTirofiban and sirolimus-eluting stent vs abciximab and bare-metal stent for acute myocardial infarction : a randomized trial. JAMA. 2005 May 4 ;293(17) :2109-17. doi : 10.1001/jama.293.17.2109
14. Windecker S, Remondino A, Eberli FR, Jüni P, Räber L, Wenaweser P, Togni M, Billinger M, Tüller D, Seiler C, Roffi M, Corti R, Sütsch G, Maier W, Lüscher T, Hess OM, Egger M, Meier B. Sirolimus-eluting and paclitaxel-eluting stents for coronary revascularization.N Engl J Med. 2005 Aug 18 ;353(7) :653-62. doi : 10.1056/NEJMoa051175. Epub 2005 Aug 16
15. Dibra A, Kastrati A, Mehilli J, Pache J, Schühlen H, von Beckerath N, Ulm K, Wessely R, Dirschinger J, Schömig A ; ISAR-DIABETES Study Investigators. Paclitaxel-eluting or sirolimus-eluting stents to prevent restenosis in diabetic patients.

N Engl J Med. 2005 Aug 18 ;353(7) :663-70. doi : 10.1056/NEJMoa044372. Epub 2005 Aug 16

16. Goy JJ, Stauffer JC, Siegenthaler M, Benoît A, Seydoux C. A prospective randomized comparison between paclitaxel and sirolimus stents in the real world of interventional cardiology : the TAXi trial. J Am Coll Cardiol. 2005 Jan 18 ;45(2) :308-11. doi: 10.1016/j.jacc.2004.10.062
17. Holmes DR Jr, Leon MB, Moses JW et al. Analysis of 1-year clinical outcomes in the SIRIUS trial : a randomized trial of a sirolimus-eluting stent versus a standard stent in patients at high risk for coronary restenosis. Circulation. 2004 Feb 10 ;109(5):634-40. doi: 10.1161/01.CIR.0000112572.57794.22
18. Stone GW, Ellis SG, Cox DA, Hermiller J, O'Shaughnessy C, Mann JT, Turco M, Caputo R, Bergin P, Greenberg J, Popma JJ, Russell ME ; TAXUS-IV Investigators. One-year clinical results with the slow-release, polymer-based, paclitaxel-eluting TAXUS stent : the TAXUS-IV trial. Circulation. 2004 Apr 27 ;109(16):1942-7. Doi : 10.1161/01.CIR.0000127110.49192.72. Epub 2004 Apr 12
19. Morice MC, Serruys PW, Sousa JE, Fajadet J, Ban Hayashi E, Perin M, Colombo A, Schuler G, Barragan P, Guagliumi G, Molnàr F, Falotico R; RAVEL Study Group. Randomized Study with the Sirolimus-Coated Bx Velocity Balloon-Expandable Stent in the Treatment of Patients with de Novo Native Coronary Artery Lesions. A randomized comparison of a sirolimus-eluting stent with a standard stent for coronary revascularization. N Engl J Med. 2002 Jun 6 ;346(23) :1773-80. doi : 10.1056/NEJMoa012843

Supplementary Reference Table S2

1. Jakobsen L, Christiansen EH, Maeng M, Hansen KN, Kristensen SD, Bøtker HE, Terkelsen CJ, Jensen SE, Raungaard B, Madsen M, Lassen JF, Jensen LO.Final five-year outcomes after implantation of biodegradable polymer-coated biolimus-eluting stents versus durable polymer-coated sirolimus-eluting stents. EuroIntervention. 2017 Dec 20 ;13(11) :1336-1344. doi: 10.4244/EIJ-D-17-00434
2. Raungaard B, Jensen LO, Tilsted HH, Christiansen EH, Maeng M, Terkelsen CJ, Krusell LR, Kaltoft A, Kristensen SD, Bøtker HE, Thuesen L, Aarøe J, Jensen SE, Villadsen AB, Thayssen P, Veien KT, Hansen KN, Junker A, Madsen M, Ravkilde J, Lassen JF; Scandinavian Organization for Randomized Trials with Clinical Outcome (SORT OUT).Zotarolimus-eluting durable-polymer-coated stent versus a biolimus-eluting biodegradable-polymer-coated stent in unselected patients undergoing percutaneous coronary intervention (SORT OUT VI): a randomised non-inferiority trial. Lancet. 2015 Apr 18 ;385(9977) :1527-35. doi: 10.1016/S0140-6736(14)61794-3. Epub 2015 Jan 16.
3. Smits PC, Vlachojannis GJ, McFadden EP, Royaards KJ, Wassing J, Joesoef KS, van Mieghem C, van de Ent M. Final 5-Year Follow-Up of a Randomized Controlled Trial of Everolimus- and Paclitaxel-Eluting Stents for Coronary Revascularization in Daily Practice : The COMPARE Trial (A Trial of Everolimus-Eluting Stents and Paclitaxel Stents for Coronary Revascularization in Daily Practice). JACC Cardiovasc Interv. 2015 Aug 17 ;8(9) :1157-1165. doi: 10.1016/j.jcin.2015.03.028. Epub 2015 Jul 22
4. Iqbal J, Serruys PW, Silber S, Kelbaek H, Richardt G, Morel MA, Negoita M, Buszman PE, Windecker S.Comparison of zotarolimus- and everolimus-eluting coronary stents: final 5-year report of the RESOLUTE all-comers trial.Circ Cardiovasc Interv. 2015 Jun;8(6):e002230. doi: 10.1161/CIRCINTERVENTIONS.114.002230
5. Natsuaki M, Kozuma K, Morimoto T, Kadota K, Muramatsu T, Nakagawa Y, Akasaka T, Igarashi K, Tanabe K, Morino Y, Ishikawa T, Nishikawa H, Awata M, Abe M, Okada H, Takatsu Y, Ogata N, Kimura K, Urasawa K, Tarutani Y, Shiode N, Kimura T. Final 3-Year Outcome of a Randomized Trial Comparing Second-Generation Drug-Eluting Stents Using Either Biodegradable Polymer or Durable Polymer : NOBORI Biolimus-Eluting Versus XIENCE/PROMUS Everolimus-Eluting Stent Trial. Circ Cardiovasc Interv. 2015 Oct ;8(10) : e002817. doi : 10.1161/CIRCINTERVENTIONS.115.002817
6. Maeng M, Tilsted HH, Jensen LO, Krusell LR, Kaltoft A, Kelbæk H, Villadsen AB, Ravkilde J, Hansen KN, Christiansen EH, Aarøe J, Jensen JS, Kristensen SD, Bøtker HE, Thuesen L, Madsen M, Thayssen P, Sørensen HT, Lassen JF. Differential clinical outcomes after 1 year versus 5 years in a randomised comparison of zotarolimus-

eluting and sirolimus-eluting coronary stents (the SORT OUT III study): a multicentre, open-label, randomised superiority trial. Lancet. 2014 Jun 14 ;383(9934) :2047-2056. doi : 10.1016/S0140-6736(14)60405-0. Epub 2014 Mar 14.

7. Di Lorenzo E, Sauro R, Varricchio A, Capasso M, Lanzillo T, Manganelli F, Carbone G, Lanni F, Pagliuca MR, Stanco G, Rosato G, Suryapranata H, De Luca G.
Randomized comparison of everolimus-eluting stents and sirolimus-eluting stents in patients with ST elevation myocardial infarction : RACES-MI trial. JACC Cardiovasc Interv. 2014 Aug;7(8):849-56. doi: 10.1016/j.jcin.2014.02.016
8. Serruys PW, Farooq V, Kalesan B, de Vries T, Buszman P, Linke A, Ischinger T, Klauss V, Eberli F, Wijns W, Morice MC, Di Mario C, Corti R, Antoni D, Sohn HY, Eerdmans P, Rademaker-Havinga T, van Es GA, Meier B, Jüni P, Windecker S.Improved safety and reduction in stent thrombosis associated with biodegradable polymer-based biolimus-eluting stents versus durable polymer-based sirolimus-eluting stents in patients with coronary artery disease: final 5-year report of the LEADERS (Limus Eluted From A Durable Versus ERodable Stent Coating) randomized, noninferiority trial.JACC Cardiovasc Interv. 2013 Aug ;6(8) :777-89. doi : 10.1016/j.jcin.2013.04.011
9. Jensen LO, Thayssen P, Hansen HS, Christiansen EH, Tilsted HH, Krusell LR, Villadsen AB, Junker A, Hansen KN, Kaltoft A, Maeng M, Pedersen KE, Kristensen SD, Bøtker HE, Ravkilde J, Sanchez R, Aarøe J, Madsen M, Sørensen HT, Thuesen L, Lassen JF; Scandinavian Organization for Randomized Trials With Clinical Outcome IV (SORT OUT IV) InvestigatorsRandomized comparison of everolimus-eluting and sirolimus-eluting stents in patients treated with percutaneous coronary intervention: the Scandinavian Organization for Randomized Trials with Clinical Outcome IV (SORT OUT IV). Circulation. 2012 Mar 13 ;125(10) :1246-55. doi :10.1161/CIRCULATIONAHA.111.063644. Epub 2012 Feb 3
10. Kandzari DE, Mauri L, Popma JJ, Turco MA, Gurbel PA, Fitzgerald PJ, Leon MB.
Late-term clinical outcomes with zotarolimus- and sirolimus-eluting stents. 5-year follow-up of the ENDEAVOR III (A Randomized Controlled Trial of the Medtronic Endeavor Drug [ABT-578] Eluting Coronary Stent System Versus the Cypher Sirolimus-Eluting Coronary Stent System in De Novo Native Coronary Artery
11. Stone GW, Teirstein PS, Meredith IT, Farah B, Dubois CL, Feldman RL, Dens J, Hagiwara N, Allocco DJ, Dawkins KD ; PLATINUM Trial Investigators A prospective, randomized evaluation of a novel everolimus-eluting coronary stent : the PLATINUM (a Prospective, Randomized, Multicenter Trial to Assess an Everolimus-Eluting Coronary

Stent System [PROMUS Element] for the Treatment of Up to Two de Novo Coronary Artery Lesions) trial. J Am Coll Cardiol. 2011 Apr 19 ;57(16):1700-8. doi : 10.1016/j.jacc.2011.02.016. Epub 2011 Apr 4

12. Leon MB, Mauri L, Popma JJ, Cutlip DE, Nikolsky E, O'Shaughnessy C, Overlie PA, McLaurin BT, Solomon SL, Douglas JS Jr, Ball MW, Caputo RP, Jain A, Tolleson TR, Reen BM 3rd, Kirtane AJ, Fitzgerald PJ, Thompson K, Kandzari DE ; ENDEAVOR IV Investigators.A randomized comparison of the Endeavor zotarolimus-eluting stent versus the TAXUS paclitaxel-eluting stent in de novo native coronary lesions 12-month outcomes from the ENDEAVOR IV trial. J Am Coll Cardiol. 2010 Feb 9;55(6):543-54. doi: 10.1016/j.jacc.2009.08.067.
13. Kereiakes DJ, Sudhir K, Hermiller JB, Gordon PC, Ferguson J, Yaqub M, Sood P, Su X, Yakubov S, Lansky AJ, Stone GW.Comparison of everolimus-eluting and paclitaxel-eluting coronary stents in patients undergoing multilesion and multivessel intervention: the SPIRIT III (A Clinical Evaluation of the Investigational Device XIENCE V Everolimus Eluting Coronary Stent System [EECSS] in the Treatment of Subjects With De Novo Native Coronary Artery Lesions) and SPIRIT IV (Clinical Evaluation of the XIENCE V Everolimus Eluting Coronary Stent System in the Treatment of Subjects With De Novo Native Coronary Artery Lesions) randomized trials. JACC Cardiovasc Interv. 2010 Dec ;3(12):1229-39. Doi : 10.1016/j.jcin.2010.09.014
14. Byrne RA, Kastrati A, Kufner S, Massberg S, Birkmeier KA, Laugwitz KL, Schulz S, Pache J, Fusaro M, Seyfarth M, Schömig A, Mehilli J; Intracoronary Stenting and Angiographic Results: Test Efficacy of 3 Limus-Eluting Stents (ISAR-TEST-4) Investigators.Randomized, non-inferiority trial of three limus agent-eluting stents with different polymer coatings: the Intracoronary Stenting and Angiographic Results: Test Efficacy of 3 Limus-Eluting Stents (ISAR-TEST-4) Trial. Eur Heart J. 2009 Oct ;30(20) :2441-9. doi : 10.1093/eurheartj/ehp352. Epub 2009 Aug 30
15. Nikolsky E, Lansky AJ, Sudhir K, Doostzadeh J, Cutlip DE, Piana R, Su X, White R, Simonton CA, Stone GW. SPIRIT IV trial design : a large-scale randomized comparison of everolimus-eluting stents and paclitaxel-eluting stents in patients with coronary artery disease. Am Heart J. 2009 Oct ;158(4) :520-526.e2. doi : 10.1016/j.ahj.2009.07.025. Epub 2009 Aug 26.
16. Camenzind E, Wijns W, Mauri L, Boersma E, Parikh K, Kurowski V, Gao R, Bode C, Greenwood JP, Gershlick A, O'Neill W, Serruys PW, Jorissen B, Steg PG; PROTECT Steering Committee and Investigators.Rationale and design of the Patient Related OuTcomes with Endeavor versus Cypher stenting Trial (PROTECT): randomized controlled trial comparing the incidence of stent thrombosis and clinical events after sirolimus or zotarolimus drug-eluting stent implantation.Am Heart J. 2009 Dec;158(6):902-909.e5. doi: 10.1016/j.ahj.2009.10.002

17. Garg S, Serruys P, Onuma Y, Dorange C, Veldhof S, Miquel-Hébert K, Sudhir K, Boland J, Huber K, Garcia E, te Riele JA ; SPIRIT II Investigators.3-year clinical follow-up of the XIENCE V everolimus-eluting coronary stent system in the treatment of patients with de novo coronary artery lesions : the SPIRIT II trial (Clinical Evaluation of the Xience V Everolimus Eluting Coronary Stent System in the Treatment of Patients with de novo Native Coronary Artery Lesions). JACC Cardiovasc Interv. 2009 Dec ;2(12) :1190-8. doi : 10.1016/j.jcin.2009.10.002
18. Fajadet J, Wijns W, Laarman GJ, Kuck KH, Ormiston J, Münzel T, Popma JJ, Fitzgerald PJ, Bonan R, Kuntz RE ; ENDEAVOR II Investigators Randomized, double-blind, multicenter study of the Endeavor zotarolimus-eluting phosphorylcholine-encapsulated stent for treatment of native coronary artery lesions : clinical and angiographic results of the ENDEAVOR II trial. Circulation. 2006 Aug 22 ;114(8) :798-806. doi : 10.1161/CIRCULATIONAHA.105.591206. Epub 2006 Aug 14 Lesions). JACC Cardiovasc Interv. 2011 May ;4(5) :543-50. doi : 10.1016/j.jcin.2010.12.014.
19. Chevalier B, Serruys PW, Silber S, Garcia E, Suryapranata H, Hauptmann K, Wijns W, Schuler G, Fath-Ordoubadi F, Worthley S, Thuesen L, Meredith I, Bressers M, Nagai H, Paunovic D. Randomised comparison of Nobori, biolimus A9-eluting coronary stent with a Taxus(R), paclitaxel-eluting coronary stent in patients with stenosis in native coronary arteries : the Nobori 1 trial. EuroIntervention. 2007 Feb ;2(4) :426-34
20. Smits PC, Hofma S, Togni M, Vázquez N, Valdés M, Voudris V, Slagboom T, Goy JJ, Vuillomenet A, Serra A, Nouche RT, den Heijer P, van der Ent M. Abluminal biodegradable polymer biolimus-eluting stent versus durable polymer everolimus-eluting stent (COMPARE II) : a randomised, controlled, non-inferiority trial. Lancet. 2013 Feb 23 ;381(9867) :651-60. doi : 10.1016/S0140-6736(12)61852-2. Epub 2013 Jan 30.
